# 18q Deletion (de Grouchy Syndrome) in Rural Romania: A Case Report and Healthcare System Challenges

**DOI:** 10.3390/reports8020084

**Published:** 2025-06-01

**Authors:** Mona Irina Matei, Raluca Maria Vlad

**Affiliations:** 1 “Alexandru Obregia” Psychiatry Clinical Hospital, 041914 Bucharest, Romania; 2 Department of Paediatrics, “Carol Davila” University of Medicine and Pharmacy, 050474 Bucharest, Romania; raluca.vlad@umfcd.ro; 3 “Grigore Alexandrescu” Emergency Children’s Hospital, 011743 Bucharest, Romania

**Keywords:** de Grouchy syndrome, 18q deletion, genetic testing, developmental delay, multidisciplinary care, rural healthcare

## Abstract

This case study presents the long-term management of a 14-year-old male diagnosed with 18q deletion syndrome, also known as de Grouchy Syndrome, highlighting the challenges of treating rare chromosomal disorders in rural Romania. **Background and Clinical Significance**: 18q deletion syndrome, also known as de Grouchy syndrome, is a chromosomal disorder caused by the deletion of a part of the long arm of chromosome 18. This syndrome is seen in one out of 10,000 live births. The main features of the syndrome are short stature, hearing loss, hypotonia, mental retardation, endocrine disorders, and autoimmunity. **Case Presentation**: The patient’s condition was initially suspected at birth due to abnormal features and was later confirmed through genetic testing, revealing a 46,XY,del(18) karyotype. Key clinical features include craniofacial dysmorphism, delayed growth, congenital cardiac anomalies, developmental delay, severe neurological impairment, and multiple comorbidities such as endocrine dysfunction, dental anomalies, and orthopedic deformities. Despite early interventions such as cardiac surgery, the patient’s management has been challenged by limited access to specialized care. **Conclusions**: The case underscores the importance of timely genetic testing, early multidisciplinary care, and the role of family support in managing complex disorders. This report also addresses the gaps in healthcare accessibility in rural settings and emphasizes the need for improved infrastructure and genetic services. By comparing this case with the existing literature, the study explores the variability in clinical presentations of 18q deletion syndrome and advocates for more precise genetic testing to better understand its phenotypic spectrum. The patient’s ongoing challenges with medical and socio-economic factors emphasize the critical need for coordinated care and family support in managing rare genetic conditions.

## 1. Introduction and Clinical Significance

18q deletion syndrome, also known as de Grouchy syndrome, is a chromosomal disorder caused by the deletion of a part of the long arm of chromosome 18. This syndrome is seen in one out of 10,000 live births. The main features of the syndrome are short stature, hearing loss, hypotonia, mental retardation, endocrine disorders, and autoimmunity [1]. It was first reported by De Grouchy in 1964 [2]. Chromosome 18q syndrome usually appears to result from spontaneous (de novo) errors very early during embryonic development that occur sporadically [3]. The deletion can occur at various points along the q arm, leading to varying degrees of phenotypic expression. Caring for these patients is a multidisciplinary challenge.

In the vast domain of pediatrics, it is easy to become captivated by the medical complexities of a case, viewing it as a puzzle to be solved. However, each case represents more than just a collection of symptoms and diagnoses. The case presented is the story of a male patient whose life has been profoundly impacted by a rare genetic condition, and also the story of a family whose dedication and resilience have been tested time and time again.

In Romania, as in many other parts of the world, families of children with rare genetic disorders often encounter significant obstacles in accessing appropriate care. The healthcare system may be fragmented, and families may struggle to understand the complex network of specialists and treatments required. This article highlights the gap between the medical care the patient needed from birth and the care he received, due to the limited availability of certain medical specialties in rural areas.

Furthermore, it aims to provide a comprehensive overview of 18q deletion syndrome through a representative case, while reminding us of the importance of empathy and compassion in medicine. Since 2010, the patient has been admitted to “Grigore Alexandrescu” Emergency Children Hospital more than 70 times, with an average duration of 2 weeks/admission and an average cost/hospitalization of 1200–1400 euros. This has had a tremendous impact on both his family’s life and the healthcare system.

## 2. Case Presentation

The authors present the case of a male patient born in 2010. His mother resided in a rural area of Romania, and she did not receive any form of prenatal care and evaluation. Romania’s maternal and child health outcomes have improved significantly in the past decade. However, reproductive outcomes remain poor for women in rural areas, minority ethnic groups, and women of low socioeconomic status [4].

The first suspicion of a genetic condition was raised by neonatologists at birth, based on the presence of abnormal features. However, it was not until he reached two months of age, when his parents and caregivers observed delays in meeting typical developmental milestones, that further investigations were initiated. Upon his initial pediatric assessment, he exhibited peculiar facial features, signs of developmental delay and growth retardation. His weight and length were both below the 5th percentile for his age, and he displayed hypotonia, with poor muscle tone and weak reflexes. A cardiac murmur was described, and he was diagnosed with Tetralogy of Fallot.

**Figure 1:**
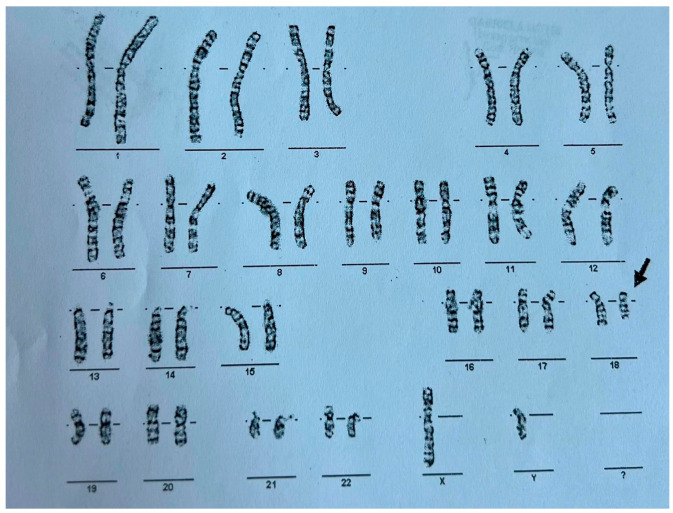
G-Banded karyotype of the patient exhibiting a deletion on the long arm of chromosome 18–46 xy, del(18).

This constellation of symptoms and features prompted a request for genetic testing. A G-banded karyotype analysis was performed in November 2012, revealing a 46,XY,del(18) karyotype indicative of 18q deletion syndrome—also known as de Grouchy Syndrome— as shown in Figure 1. Further genetic investigations to determine the exact region of chromosome 18q were not performed. According to the literature, every unrelated person with chromosome 18q deletion has a unique region of hemizygosity and this region can involve almost any region of 18q, including between 1 and 101 genes (30 Mb of DNA), with most individuals having terminal deletions [5]. While conventional karyotyping remains a valuable first-line tool for identifying major chromosomal abnormalities, it offers only a broad overview of structural changes and cannot precisely define the size or gene content of a deletion. In this case, although karyotyping successfully identified the 18q deletion, supplementary investigations such as microarray analysis could have provided a more detailed characterization of the chromosomal abnormality. Unfortunately, in our healthcare setting, the costs associated with supplementary genetic investigations, such as microarray analysis, must often be covered by the patient’s family. Due to financial constraints, many families were unable to access these additional diagnostic resources.

Following his initial diagnosis, the patient’s medical journey was marked by frequent hospitalizations and ongoing medical interventions.

### 2.1. Clinical Presentation and Complex Multidisciplinary Management

The patient is now 14 years old. He presents craniofacial dysmorphism, delayed growth, cardiovascular and orthopedic malformations, dental and Ear, Nose, Throat (ENT) anomalies, severe neurological disability, and mental retardation.

#### 2.1.1. Craniofacial Dysmorphism

**Figure 2:**
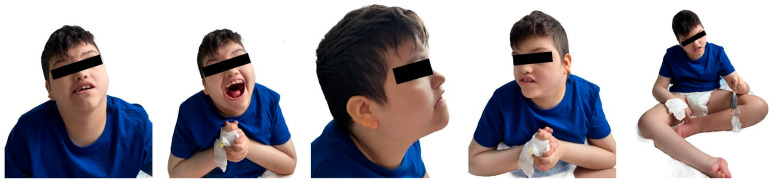
Craniofacial dysmorphism, dental and orthopedic anomalies.

The patient’s physical examination revealed microcephaly, low-set, malformed ears, and a flat nasal bridge, a “carp-shaped” mouth, and prognathism, as depicted in Figure 2.

#### 2.1.2. Growth Retardation and Nutritional Challenges

The patient’s growth remained suboptimal throughout his childhood. Feeding difficulties, including oromotor dysfunction and recurrent respiratory infections, resulted in serious nutritional challenges. To address his growth failure, a pediatric nutritionist could have created a tailored dietary plan that included calorie-dense feeds, supplements, and periodic assessments of his nutritional status, but for most of his childhood this was not available.

At present, he has good somatic growth parameters: weight = 40 kg (z-score = −0.61), height = approximately 1.50 m (z-score = −1). However, precise measurement is difficult to severe articular deformities (he cannot assume an orthostatic position). His BMI is 17.78 (pc 22, z-score = −0.75). However, he exhibits delayed puberty, with infantile external genitalia and minimal, sparse pubic hair, consistent with Tanner stage 2.

#### 2.1.3. Congenital Cardiac Malformation

**Figure 3:**
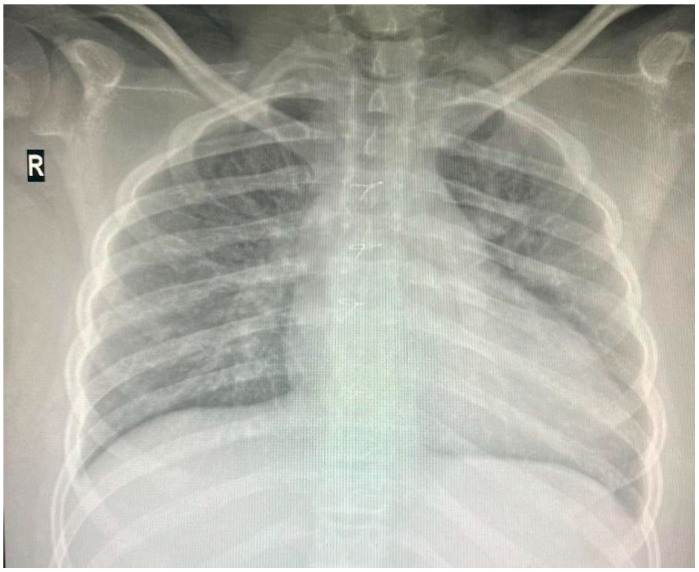
Thoracic X-ray: cardiomegaly, boot-shaped heart.

Individuals with 18q deletion syndrome present with various clinical characteristics, including cardiac anomalies. Nonetheless, genotype–phenotype correlations for cardiac anomalies in 18q deletion syndrome have rarely been reported [6] The patient was diagnosed with Tetralogy of Fallot, a congenital heart defect characterized by four anatomical abnormalities: ventricular septal defect (VSD), pulmonary stenosis, right ventricular hypertrophy, and an overriding aorta. He underwent successful corrective surgery in Germany at the age of one and has received infective endocarditis prophylaxis ever since. Since then, he has received biannual pediatric cardiology consultations at “Grigore Alexandrescu” Emergency Children’s Hospital. His latest cardiac evaluation described a grade II diastolic murmur at the left parasternal area, and the cardiac ultrasound showed no residual shunts but moderate pulmonary regurgitation. Cardio-pulmonary x-ray at the age of 14 is depicted in Figure 3.

#### 2.1.4. Neurological Disability

The patient began experiencing tonic seizures at the age of three, leading to a diagnosis of symptomatic epilepsy. His seizures are managed with antiepileptic medication, and electroencephalography (EEG) should be performed periodically. In addition to epilepsy, he exhibits signs of neurodevelopmental delay, including profound intellectual disability and profoundly delayed speech development, currently limited to only short phonemes and cries. He undergoes periodic evaluations; however, due to the limited availability of pediatric neurologists in his living area, his assessments have been less frequent than recommended. Consequently, his treatment has not been consistently adjusted in response to the progression of his symptoms. He is currently receiving treatment for epilepsy with Valproate, and his most recent EEG (June 2024) revealed no abnormalities. Typical features cited in the literature include poor cerebral myelination, reduced intellectual functioning, and developmental delay [7].

#### 2.1.5. Psychiatric Aspects

The patient is non-verbal and manifests symptoms suggestive of ADHD, such as impatience, impulsivity, and sporadic episodes of physical auto- and hetero-aggressivity. He exhibited hyperkinetic behavior, screaming, crying. He spends hours on end engaged in automatic, repetitive behavior (playing with keys or clicking pens). The neuropsychiatric phenotype of 18q del is not well documented and includes disorganized and disinhibited actions as well as language difficulties [8].

#### 2.1.6. Dental and ENT Anomalies

Dental involvement in patients with deletion syndrome of the long arm of chromosome 18 is poorly documented in the literature. The hospital regimen appears to be the standard criterion for the management of these patients. A case report of an 8-year-old boy with chromosome 18q deletion describes the following stomatological management: the oral cavity revealed a destructive carious lesion of the lower right second deciduous molar and the need to perform a frenectomy due to the short lingual frenulum. Given the complex management of the patient, it was necessary to carry out surgical procedures in the operating room. Significant dental issues were present, including delayed eruption of teeth, multiple caries, and malocclusion [9]. In our case, during the current hospital admission for an infection of unknown origin (one of the many in a row), a persistent dental source was suspected, and he was referred to a pediatric dentist. He received his first dental consultation at the age of 14. He underwent major dental surgery in June 2024. The procedure was done under general anesthesia, due to the complexity of his dental problems and his inability to cooperate with procedures in a conventional setting. There were no complications following the surgery. All abscesses were drained and supernumerary teeth were extracted; subsequent antibiotic treatment was prescribed. No further repeated episode of fever followed.

18q del patients’ dental health requires ongoing attention, with frequent dental cleanings, fluoride treatments, and restorative work to manage caries and malocclusions. This care should be coordinated by a pediatric dentist familiar with the challenges posed by patients with intellectual disabilities and complex medical histories. The use of general anesthesia for dental procedures is almost always necessary to ensure that all necessary work can be completed safely.

The 18q syndrome is associated with hearing impairment in 50–80% of cases. The hearing loss may be sensorineural or conductive. A high proportion of cases are associated with narrow external auditory canals [10]. In our case, preauricular appendages are present on the right ear and the patient has not been diagnosed with hearing loss (normal acoustic impedance).

#### 2.1.7. Orthopedic Malformation and Severe, Progressive Limb Deformity

**Figure 4:**
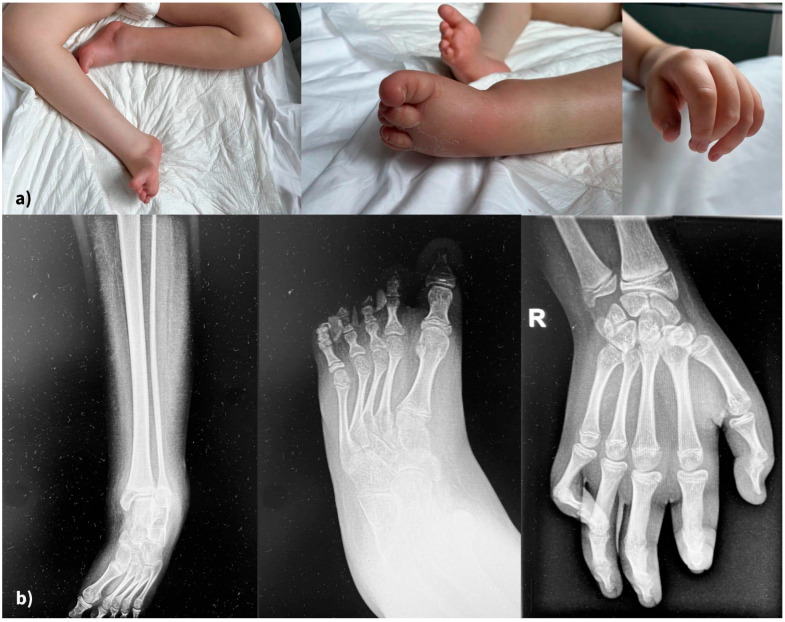
(**a**) Severe, irreversible limb deformities (clinical aspects), (**b**) X-rays of lower and upper limbs.

Skeletal abnormalities were another major concern. Figure 4 displays the clinical (4a) and radiological (4b) aspects of his limb deformity. The management of our patient’s orthopedic malformation should have focused since early infancy on maintaining mobility and preventing further deformities. At present, he has multiple joint contractures, scoliosis, and short stature due to delayed bone growth. He was recommended a series of procedures through the years, including physiotherapy and corrective bracing. Unfortunately, this kind of medical service was not readily available to his family.

Recent genomic studies have linked genes located on the 18 q chromosome to adolescent idiopathic scoliosis and pectus excavatum, both present in our patient [11].

#### 2.1.8. Endocrine Manifestations

Our patient has subclinical primary hypothyroidism (normal ATPO, normal freeT4, high TSH = 12 IU/mL). His latest endocrine consultation concluded the following: Phenotypic facial features. Height could not be measured due to bilateral lower limb malformations. Severe developmental delay. Dystrophic dentition. Significant scrotal edema; testicular volume could not be assessed bilaterally. A close monitoring of his 25-OH vitamin D and PTH levels was advised, and it was also suggested that TSH elevation may be secondary to his valproic acid treatment for epilepsy. The endocrinology evaluation also included assessments of his growth hormone axis with normal findings.

#### 2.1.9. Hematological Anomalies

During his admission in May 2024, his left lower limb was swollen, red, and warm to the touch, prompting an urgent hematology consultation to rule out deep vein thrombosis. But, due to the recurrent nature of the complaint, further tests on coagulation and a thrombophilia panel were performed, and genetic anomalies were found: MTHFR C677T positive heterozygous, polymorphism of the PAI-1 gene at position 675: 4G/4G (normal: 5G/5G), EPCR: A1/A3 (normal: absence of the A3 allele). According to the literature, DNA deletions such as 18q del have also been implicated in diseases of immune dysregulation, which might explain his recurrent presentations for infections of an unknown source [12].

#### 2.1.10. Socio-Economic Issues and Family Concerns

In this case, the patient’s grandmother played a pivotal role in ensuring that he receives the best possible care. She has been deeply involved in every aspect of his medical management, from attending every appointment to meticulously following the advice of his healthcare providers.

This level of involvement is, unfortunately, not always the norm. In many cases, the cooperation between healthcare providers and families can be strained due to various factors, including logistical challenges, financial constraints, and differences in understanding or prioritization of medical advice.

## 3. Discussions

This case exemplifies the complexities of managing rare chromosomal disorders, such as 18q deletion syndrome, in resource-limited settings. His management will be discussed in parallel with the recommendations from the National Organization for Rare Disorders (NORD), highlighting both successes and gaps in medical care.

As NORD emphasizes, individualized care plans are critical to addressing the unique needs of each patient [3]. The phenotypic presentation in our case, including significant neurodevelopmental delays, cardiac anomalies, and growth challenges, required a highly tailored management approach that was only partially achieved.

### 3.1. Phenotypic Variability and Genomic Studies

Partial deletions of the long arm of chromosome 18 leads to variable phenotypes, with severity determined by the size and location of the chromosomal deletion.

Common clinical features include a characteristic face, short stature, congenital aural atresia (CAA), abnormalities of the feet, and mental retardation. The presence or absence of these clinical features may depend on the size and position of the deleted region.

Using new molecular techniques such as array CGH, which allows for a more precise determination of breakpoints in cytogenetic syndromes, scientists were able to refine the critical regions for microcephaly (18q21.33), short stature (18q12.1–q12.3, 18q21.1–q21.33, and 18q22.3–q23), white matter disorders and delayed myelination (18q22.3–q23), GHD (18q22.3–q23), and CAA (18q22.3). Additionally, the overall level of mental retardation appeared to be mild in patients with deletions distal to 18q21.33 and severe in patients with deletions proximal to 18q21.31. The critical region for the ‘typical’ 18q phenotype is a region of 4.3 Mb located within 18q22.3–q23. Molecular characterization of more patients will ultimately lead to a further delineation of the critical regions and thus to the identification of candidate genes for these specific traits [13].

**Figure 5:**
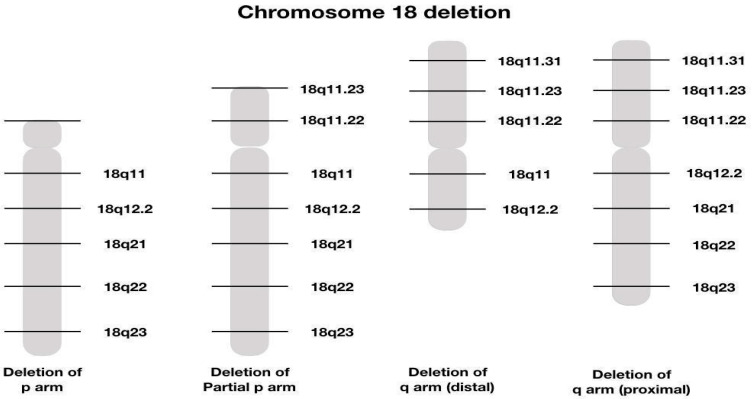
Graphical illustration of different chromosome 8 deletions [14].

Different deletions described on chromosome 18 are represented in Figure 5

### 3.2. Genetic Testing and Early Diagnosis

NORD stresses the importance of early genetic testing to confirm the diagnosis and facilitate immediate management planning [3]. In our case, this crucial step was delayed until the age of two, resulting in postponed interventions and care optimization. Earlier testing could have enabled comprehensive family counseling, developmental therapies, and early nutritional support. This was due to a lack of both access and medical information in the rural area where the family resides. Since Watson and Crick’s description of the structure of DNA, significant progress has been made in controlling congenital disorders, with most benefits accruing to industrialized areas. Developing nations have seen little advantage; during the same time frame, they underwent a substantial epidemiological transition, leading congenital disorders to become a major public health concern. The burden of congenital disorders in these lower-resource countries and rural areas is high and they need to develop medical genetic services [15].

Many concerned scientists also believe that developments in the medical application of genomics will widen the gap between the developed and the developing world. Most developing countries now urgently need to incorporate genetic approaches, as DNA diagnosis is relatively inexpensive, helps to develop skills in molecular biology, and provides a basis for developing national expertise in genomics [16].

### 3.3. Multidisciplinary Care and Long-Term Monitoring and Prognosis

Timely interventions are vital for improving outcomes in patients with 18q deletion syndrome. Our patient’s early cardiac surgery was pivotal, aligning with NORD’s recommendations for early correction of life-threatening anomalies. While he underwent life-saving cardiac surgery, it is notable that this was performed in Germany, reflecting the unavailability of advanced surgical options for complex congenital heart disease at that time in Romania. This highlights the need to develop a domestic healthcare infrastructure capable of addressing all stages of complex care locally. The ideal management of 18q deletion syndrome, as recommended by NORD, involves coordinated input from cardiologists, neurologists, endocrinologists, geneticists, and other specialists. The narrow palette of medical services in rural Romania posed a barrier to the patient’s care and each consultation had to be performed in Bucharest, which represented a substantial financial and logistical inconvenience for the family. Since joining the EU, Romania has seen improved quality of life and healthcare initiatives, including those related to pediatrics and neonatal care, but there is still a long way to go to address the major disparities in care between rural and urban areas [17].

Other aspects of his care, such as growth monitoring, annual endocrine evaluation, and nutritional support, were inconsistently addressed. Based on studies, it appears that annual thyroid testing, using TSH as a primary screening tool, is recommended. The mechanism of hypothyroidism is not yet known, and the genetic basis has not been delineated [18].

There are still concerning issues regarding collaboration between pediatric subspecialties. It will be crucial for us to move beyond the concept of “my patient” to “our patient” [19].

Given the numerous comorbidities and possible complications, there are no guarantees when it comes to life expectancy and long-term prognosis. However, we found in the literature the case of a 67-year-old woman with 18q deletion syndrome. Only three other adults with 18q del syndrome have been described. They were all from the same family. A follow-up study of this family revealed that these adults died around the age of 50 years old with pathological evidence of a neurodegenerative process [20].

### 3.4. The Family Role and Emotional Impact

The role of the family in managing a child with a rare disorder cannot be overstated. Caring for a child with a genetic disorder can be challenging and has profound effects on families. This situation is exacerbated by limited resources and underdeveloped diagnostic and treatment capabilities. Caregivers are the primary figures involved in caring for children with rare diseases including genetic disorders and their roles and responsibilities in the form of feeding, bathing, and administering medication to their children. Parents caring for children and adolescents with rare diseases fear the long-term progression of the child’s disease and the loss of their parental role [21]. These challenges place a heavy burden on caregivers, disrupting other family responsibilities. Caregivers of children with genetic disorders have been reported to experience psychological distress, receive less support, struggle financially, and have problems with maintaining employment [22]. In our case, the grandmother’s dedication to his care highlights the indispensable role of family support. While her efforts ensured that he received essential medical attention, systemic barriers, such as limited access to specialists in their rural area of residence, hindered optimal care delivery. In addition to providing top-quality medical care, pediatricians should also prioritize the mental health and emotional support of the caregivers.

A study conducted to identify the emotional impact of a genetic diagnosis on the family identified eight emotional effects: anxiety, worry about risks to children, guilt, anger, uncertainty, sadness and grief, depression, and redemptive adjustment. Two factors were identified that could modify the emotional effects: the variability of genetic diseases, which in the future could be better understood using genomic medicine, and the lack of diagnosis/inappropriate care, which unfortunately applied in our case. Despite many negative effects, results also suggest that redemptive adjustment is possible where a genetic condition is present in a family [23].

Adherence to medical advice in Romania tends to be inconsistent, with some families seeking multiple opinions without fully following through on recommended treatments. Our patient’s story, however, is a fortunate one in this regard, as his grandmother has been a steadfast advocate for his care, ensuring that he never missed an appointment and that he received the comprehensive care he needed. This dedication has been fundamental in managing his condition and highlights the critical role that family involvement plays in pediatric care. 

### 3.5. Bridging Gaps in Care

This case reveals a significant disparity between the ideal care recommended in the literature and the realities of healthcare delivery in Romania. This gap underscores how far the system is from providing optimal care for patients with complex genetic syndromes, particularly in rural regions. Furthermore, as noted, the mother did not receive any form of prenatal care—a situation that remains alarmingly common in rural areas and among families with lower education levels in urban regions. Despite the significant evolution in recent years in the medical field, many fetal conditions that can be detected in the early stages remain a social and economic burden due to a lack of diagnostic and treatment programs, especially in rural areas [24].

Multiple studies have been conducted to address this disparity, revealing several reasons: lack of medical education, with women in rural areas viewing pregnancy as a natural process which does not require medical supervision; long distance to the nearest hospital, creating economic barriers to accessing maternity care; and unaffordable transport [4].

This emphasizes the urgent need for large-scale educational programs targeting vulnerable communities to raise awareness about the importance of prenatal testing and the profound lifelong impact that genetic conditions can have on affected families. Systemic reforms are needed to address these gaps, including the establishment of local genetic testing facilities, comprehensive training programs for specialists, and increasing the capacity to perform complex surgical interventions domestically. Achieving these goals would ensure that patients like Eduard can receive the full spectrum of care without needing to seek treatment abroad.

### 3.6. Pharmacogenomics and Personalized Medicine

Personalized medicine and precision medicine present new opportunities for better patient care, but also present unique challenges [25].

For patients like ours, choosing the appropriate medicines is extremely difficult, due to multiple factors: comorbidities, response variability, and difficulty in monitoring treatment responses. Pharmacogenetics is a promising tool in this regard. With genotyping technologies now widely available and decreasing in cost, implementing pharmacogenomics into clinical practice is currently a focus in many countries worldwide. [26]. Many medical centers in the United States have implemented pharmacogenomics (PGx) programs to integrate PGx into clinical practice [27]. Many coordinated international efforts are ongoing in order to overcome the existing barriers to pharmacogenomic implementation [28].

## Data Availability

Data are contained within the article.

## Abbreviations

The following abbreviations are used in this manuscript:

GHD	growth hormone deficiency
NORD	National Organization for Rare Disorders
ENT	Ear, Nose, Throat
VSD	ventricular septal defect
EEG	electroencephalography
ADHD	attention deficit and hyperactivity disorder
ATPO	anti thyroperoxidase antibodies
freeT4	free thyroxine
TSH	thyroid stimulating hormone
PTH	parathyroid hormone
MTHFR	methylene tetrahydrofolate reductase
PAI	plasminogen activator inhibitor
EPCR	endothelial protein C receptor
PGx	pharmacogenomics
